# The Top 100 Most Cited Articles on Intrauterine Adhesion: a Bibliometric Analysis

**DOI:** 10.1007/s43032-021-00794-x

**Published:** 2021-11-15

**Authors:** Pan Gu, Waixing Li, Xingping Zhao, Dabao Xu

**Affiliations:** Department of Gynecology, Third Xiangya Hospital, Central South University, 138 Tongzipo Road, Changsha, 410013 Hunan China

**Keywords:** Intrauterine adhesion, Hysteroscopy, Hysteroscopic adhesiolysis, Bibliometric analysis

## Abstract

Bibliometric analysis is a statistical method that attempts to assess articles by their citations, analyzing their frequency and citation pattern, which subsequently gleans direction and guidance for future research. Over the past few years, articles focused on intrauterine adhesions have been published with increasing frequency. Nevertheless, little is known about the properties and qualities of this research, and no current analysis exists that has examined the progress in intrauterine adhesion research. Web of Science Core Collection, BIOSIS Citation Index, and MEDLINE database were searched to identify articles on intrauterine adhesion published from 1950 to October 2020. The 100 most cited articles were chosen to analyze citation count, citation density, authorship, theme, geographic distribution, time-related flux, level of evidence, and network analysis. An overwhelming majority of these 100 articles were published in the 2010s (35%). Citations per article ranged from 30 to 253. Chinese authors published the most papers in the top 100, followed by the USA, France, Israel, and Italy. The most salient study themes included operative hysteroscopy and adjunctive treatments for improving reproductive outcomes. The most common level of evidence was level II, and there was no statistical difference in the number of citations between the levels. The network analysis indicated that hysteroscopy, hysteroscopic adhesiolysis, infertility, and the reproductive outcome had a great degree of centrality in the 2000s and 2010s. In comparison, placental implantation had a great degree of centrality in the 2000s, and stem cell and fibrosis had a great degree of centrality in the 2010s. The value of IUA investigation has been gradually appreciated recently. Hysteroscopic adhesiolysis was continuously explored to achieve better reproductive outcome. Over time, the main focus of research has gradually shifted from complications to postoperative adjuvant treatment. Moreover, breakthrough progress is needed in underlying mechanism and early prevention of IUA.

## Introduction

Intrauterine adhesion (IUA) was first defined and reported by Asherman in 1950 [1, 2]. IUA is caused by mechanical injury or damage from infection to the basal layer of the endometrium, resulting in the formation of fibromuscular or severe connective tissue adhesion of the uterine cavity or cervical canal [3, 4]. Hypomenorrhea/amenorrhea [5], infertility[6], recurrent pregnancy loss [7], and obstetric complications [8] are common complications of IUA, and IUA may also lead to low birth weight [9]. At present, IUA remains a relatively intractable disease that seriously affects women’s reproductive prognosis and quality of life in childbearing age [6, 10].

Surgical treatment is the first choice for IUA. However, hysteroscopic adhesiolysis, the standard treatment for IUA, confers limited therapeutic benefit [11]. After hysteroscopic adhesiolysis, the conception rate is 25%, and the rate of reformation of adhesions is 20–63% [12–15]. In order to reduce the recurrence rate of IUA and improve reproductive prognosis, the application of other preventative and treatment measures for comprehensive management of adhesions after the surgery is recommended [16–18]. These measures mainly include devices to keep opposing endometrial surfaces separated, such as intrauterine device (IUD) [19]; Foley catheter balloon [20]; auto-crosslinked hyaluronic acid (ACP) gel [21–24]; and interventions to promote endometrial regeneration, such estrogen [25, 26], stem cell [27–29], stem cell exosome [30], amniotic epithelial cells [31], granulocyte colony-stimulating factor [32], platelet-rich plasma [33], and aspirin [34]. However, the efficacy of these measures is still not ideal: the reformation rate is as high as 48% [35], the conception rate is about 44.3%, and the live birth rate is 37.8% [36]. Therefore, reducing the reformation rate of IUA and increasing the postoperative conception rate and live birth rate have remained the focus of IUA research.

The current study aimed to identify the 100 most cited essays in the field of IUA, ascertain the research trends and hotspots in this area, and evaluate the research quality and properties of the most cited original papers over the past 70 years. We intend that the findings from this analysis can guide subsequent research in IUA.

## Methods

### Search Strategy

All articles were selected through a search of the Web of Science Core Collection, BIOSIS Citation Index, and MEDLINE to retrieve all articles related to IUA. Two individuals simultaneously conducted the search process to enhance the search sensitivity. The terms used for searching were as follows: Intrauterine adhesion OR Asherman Syndrome.

**Figure 1:**
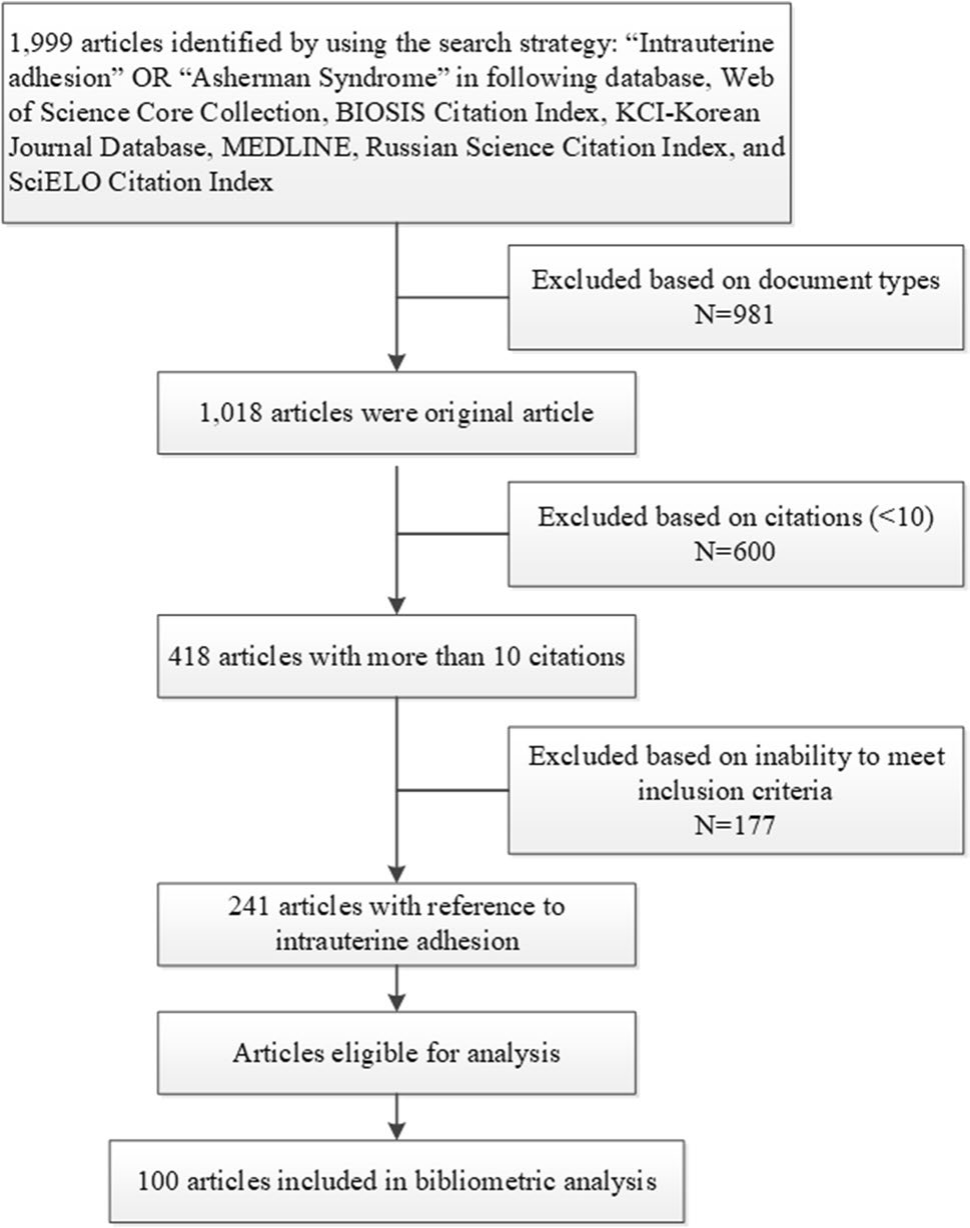
Flowchart illustrating the process of allocation of articles

The search was conducted in October 2020 and yielded a total of 1999 results. Subsequently, search results were filtered. Only original articles were included, meaning reviews, systematic reviews, meta-analyses, and guidelines were all excluded. To limit the number of screened articles, those articles that were cited fewer than 10 times were excluded. As a result, 418 articles were included for analysis. Two independent researchers reviewed the title and the abstract of the selected articles. Articles that met any of the following criteria were included: (1) basic study, animal study, and clinical trials related to any aspect of IUA; (2) the clinical therapeutic, prognostic, diagnostic, epidemiological studies of IUA; or (3) the case report data of IUA. Any disagreements between the 2 reviewers were discussed until a consensus was reached. After the title and abstract review were completed, 241 articles remained. These articles were ranked in descending order of citations, and the top 100 most cited articles were included in this analysis (Fig. 1).

### Data Extraction

Two independent, well-trained individuals reviewed all articles. The following information was listed for all articles: the journal name, publication date, first author, year of publication, geographic origin, the total number of citations of the article, overall citation rate (total citations/article age), research theme, and level of evidence (methodology has been described elsewhere [37]).

### Statistical Analysis

The Shapiro–Wilk test was used to test the distribution of individual variables for normality. Normally distributed data are presented as mean ± standard deviation. Comparison between means was performed using one-way analysis of variance (ANOVA), and post hoc testing was conducted as necessary. Time-dependent trends were tested using the Mann–Kendall trend test. Correlation between variables was performed using the Spearman rank or Pearson tests. A *P* value < 0.05 was considered to be statistically significant. Analysis was performed using SPSS Statistics 20.0 (IBM Corp., Armonk, NY, USA). UCINET for Windows, version 6.212, was used to perform the degree of centrality analysis [38].

## Results

**Figure 2:**
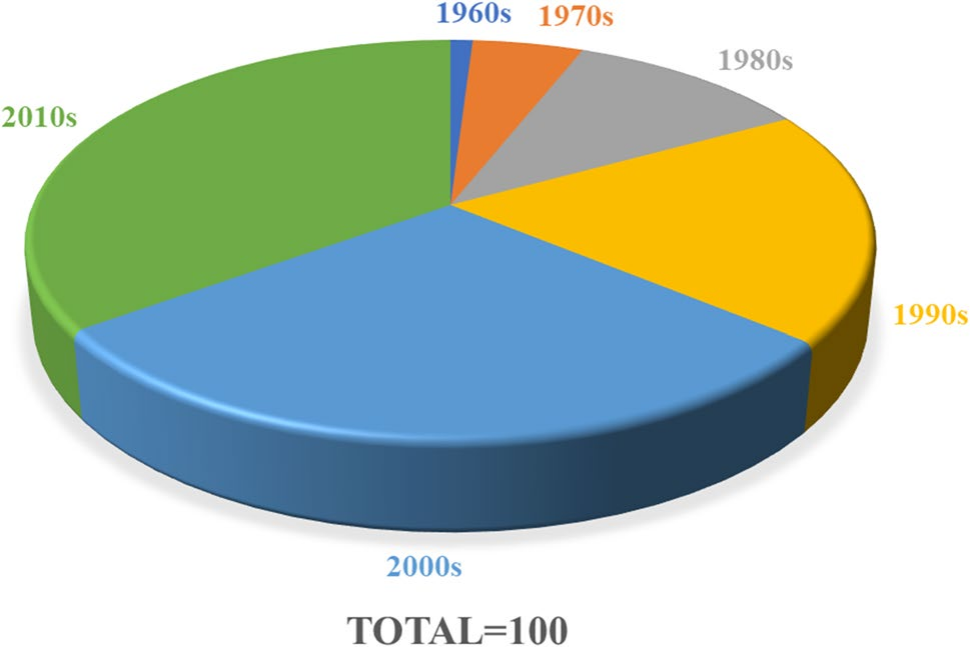
Time distribution of top 100 most-cited articles in intrauterine adhesion

**Figure 3:**
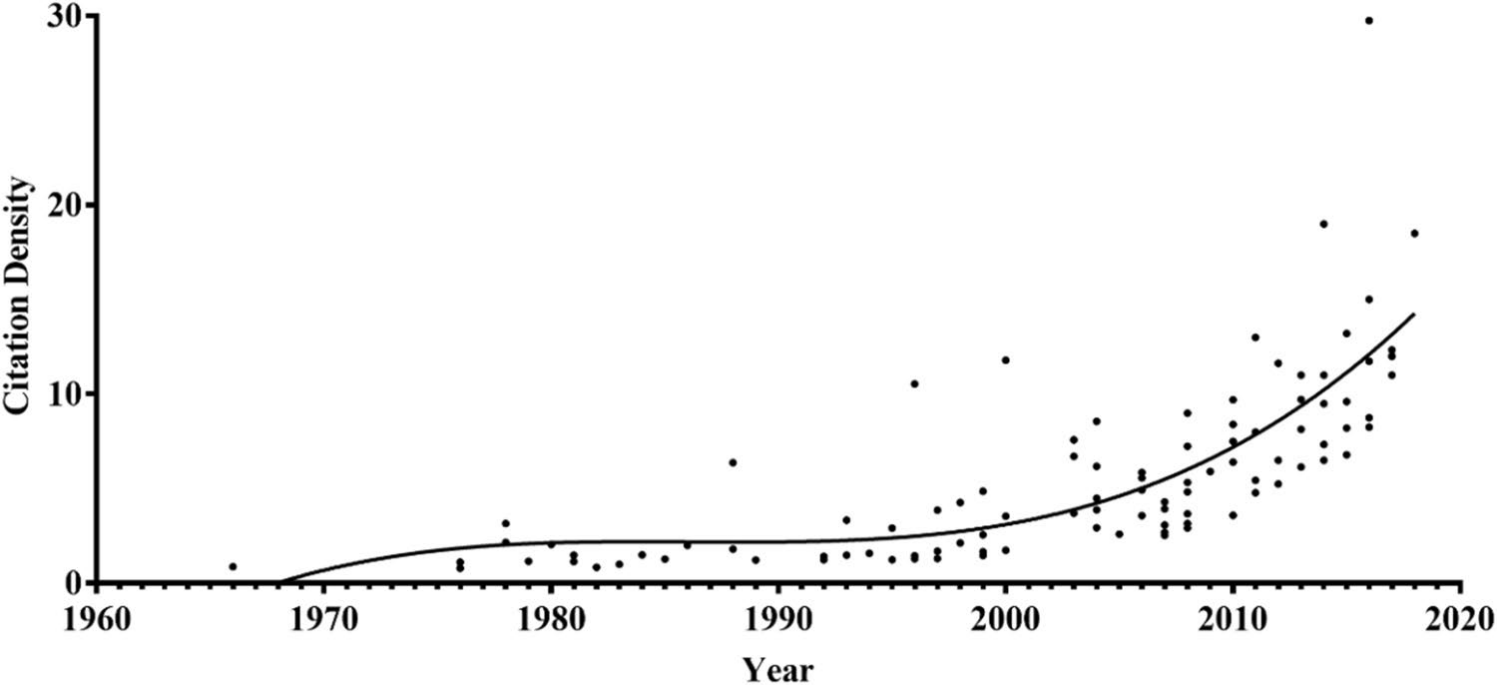
Time-dependent citation density trend of the 100 most-cited articles in intrauterine adhesion

We extracted the top 100 most cited articles in the field of IUA (listed in Table 1). Citations ranged from 30 to 253 in number, with a majority being published in the 2000s (29%) and 2010s (35%), indicating an overall trend of year-by-year increase publication (Fig. 2). The year 2008 saw the highest number of these IUA articles published (*n*=7). The number of citations was 6462 overall: 47 (0.7%) in the 1960s, 356 (5.5%) in the 1970s, 721 (11.2%) in the 1980s, 1190 (18.4%) in the 1990s, 2097 (32.5%) in the 2000s, and 2051 (31.7%) in the 2010s. The Mann–Kendall trend test showed no time-dependent trend in the publication time of articles (*P*=0.4654) but did reveal an increasing trend between the citation density and time (*P*=2.2E-16; Fig. 3). The Spearman rank analysis indicated a positive correlation between time and citation density (*r*=0.836; *P* <2.2E-16). The Shapiro–Wilk test and the Kolmogorov–Smirnov test both indicated an abnormal distribution of the citation data. The Shapiro–Wilk test indicated significant departures from normality (*P*<0.05) for all distributions tested.

**Table 1:** List of the top 100 most-cited articles in intrauterine adhesion research

Rank	Publication year	Total citation	Title	PMCID
1	1996	253	Frequency of factors associated with habitual abortion in 197 couples	8752606
2	2000	236	Diagnostic accuracy at sonohysterography, transvaginal sonography, and hysterosalpingography in patients with uterine cavity diseases	10685551
3	1988	204	Intrauterine adhesions: hysteroscopic diagnosis, classification, treatment, and reproductive outcome	3381869
4	2004	137	Effectiveness of auto-crosslinked hyaluronic acid gel in the prevention of intrauterine adhesions after hysteroscopic surgery: a prospective, randomized, controlled study	15105384
5	1978	133	Hysteroscopic management of intrauterine adhesions	637078
6	2003	129	Effectiveness of auto-cross-linked hyaluronic acid gel in the prevention of intrauterine adhesions after hysteroscopic adhesiolysis: a prospective, randomized, controlled study	12923149
7	2016	119	Autologous cell therapy with CD133+bone marrow-derived stem cells for refractory Asherman syndrome and endometrial atrophy: a pilot cohort study	27005892
8	2011	117	Endometrial regeneration using autologous adult stem cells followed by conception by in vitro fertilization in a patient with severe Asherman's syndrome	21772740
9	2003	114	A comparison of two adjunctive treatments for intrauterine adhesions following lysis	12834941
10	2014	114	Bone Marrow-Derived Stem Cell (BMDSC) transplantation improves fertility in a Murine Model of Asherman's Syndrome	24819371
11	2008	108	Factors affecting the reproductive outcome of hysteroscopic adhesiolysis for Asherman's syndrome	17681324
12	1999	102	Hysteroscopic treatment of severe Asherman's syndrome and subsequent fertility	10325268
13	2004	99	1000 office-based hysteroscopies before in vitro fertilization: feasibility and findings	15119651
14	2010	97	Reproductive outcome following hysteroscopic adhesiolysis in patients with infertility due to Asherman's syndrome	19455349
15	1998	94	Prevalence of Asherman's syndrome after secondary removal of placental remnants or a repeat curettage for incomplete abortion	9886512
16	2012	93	Comprehensive management of severe Asherman syndrome and amenorrhea	22100167
17	1978	91	Diagnostic and therapeutic hysteroscopy for traumatic intrauterine adhesions	677196
18	1993	90	Incidence of post-abortion intra-uterine adhesions evaluated by hysteroscopy--a prospective study	8473464
19	1997	89	Hysteroscopic treatment of intrauterine adhesions is safe and effective in the restoration of normal menstruation and fertility	9418714
20	2008	87	Postoperative adhesiolysis therapy for intrauterine adhesions (Asherman's syndrome)	18571166
21	2010	84	Prevalence of unsuspected uterine cavity abnormalities diagnosed by office hysteroscopy before in vitro fertilization	20570971
22	1980	82	Hysteroscopy in the evaluation of female infertility	7386525
23	2006	82	Amnion graft following hysteroscopic lysis of intrauterine adhesions	17100817
24	2006	78	Fertility after treatment of Asherman's syndrome stage 3 and 4	16962521
25	2013	77	A comparison of the intrauterine balloon, intrauterine contraceptive device and hyaluronic acid gel in the prevention of adhesion reformation following hysteroscopic surgery for Asherman syndrome: a cohort study	23932377
26	2010	75	Human amnion as a temporary biologic barrier after hysteroscopic lysis of severe intrauterine adhesions: a pilot study	20576472
27	1995	73	Reproductive outcome following hysteroscopic management of intrauterine septum and adhesions	8567788
28	2004	72	Live delivery rates in subfertile women with Asherman's syndrome after hysteroscopic adhesiolysis using the resectoscope or the Versapoint system	15169591
29	2011	72	Hysteroscopic management of residual trophoblastic tissue is superior to ultrasound-guided curettage	22024264
30	2000	71	Predictive value of transvaginal sonography performed before routine diagnostic hysteroscopy for evaluation of infertility	10685552
31	2006	69	Reduction of postoperative adhesions with an auto-crosslinked hyaluronan gel in gynecological laparoscopic surgery: a blinded, controlled, randomized, multicentre study	16439505
32	1986	68	Severe obstetric complications after aggressive treatment of Asherman syndrome	3703411
33	2013	68	Optimal waiting period for subsequent fertility treatment after various hysteroscopic surgeries	23433831
34	2014	66	Etiology, treatment, and reproductive prognosis of women with moderate-to-severe intrauterine adhesions	24598346
35	2015	66	Human CD133(+) bone marrow-derived stem cells promote endometrial proliferation in a murine model of Asherman syndrome	26384164
36	2009	65	Uterine synechiae after bipolar hysteroscopic resection of submucosal myomas in patients with infertility	18937941
37	2008	64	Efficiency and pregnancy outcome of serial intrauterine device-guided hysteroscopic adhesiolysis of intrauterine synechiae	18774563
38	2010	64	Fertility and pregnancy following pelvic arterial embolization for postpartum hemorrhage	19832826
39	2003	63	A prospective comparative study between hysterosalpingography and hysteroscopy in the detection of intrauterine pathology in patients with infertility	12696625
40	2004	62	Diagnostic value of hysterosalpingography in the detection of intrauterine abnormalities: A comparison with hysteroscopy	15505312
41	2016	60	Autologous menstrual blood-derived stromal cells transplantation for severe Asherman's syndrome	27664218
42	1981	58	Gestational outcome following hysteroscopic lysis of adhesions	6269905
43	1988	58	Endometrial abnormalities: evaluation with transvaginal sonography	3275446
44	2008	58	Office hysteroscopic early lysis of intrauterine adhesion after transcervical resection of multiple apposing submucous myomas	17686478
45	2013	57	Role of angiogenesis in the endometrial repair of patients with severe intrauterine adhesion	23826415
46	2014	57	Effect of stem cell application on Asherman syndrome, an experimental rat model	24974357
47	2007	56	Genital tuberculosis in Indian infertility patients	17362955
48	1984	54	Comparison of diagnostic accuracy of laparoscopy, hysteroscopy, and hysterosalpingography in evaluation of female infertility	6232154
49	1999	54	Transvaginal sonohysterographic evaluation of intrauterine adhesions	10064410
50	2012	52	The effect of collagen-binding vascular endothelial growth factor on the remodeling of scarred rat uterus following full-thickness injury	22136717
51	2007	51	Intrauterine adhesions as a risk factor for failed first-trimester pregnancy termination	17900447
52	2006	50	Successful use of vaginal sildenafil citrate in two infertility patients with Asherman's syndrome	16724891
53	1976	49	Obstetric complications after treatment of intrauterine synechiae (Asherman's syndrome)	934560
54	2011	49	Efficacy of a polyethylene oxide-sodium carboxymethylcellulose gel in prevention of intrauterine adhesions after hysteroscopic surgery	21777835
55	1979	48	Hysteroscopy in 100 patients	437163
56	2015	48	Randomized, controlled trial comparing the efficacy of intrauterine balloon and intrauterine contraceptive device in the prevention of adhesion reformation after hysteroscopic adhesiolysis	25936237
57	1966	47	The pathology of postcurettage intrauterine adhesions	5928449
58	1998	47	Myometrial scoring: a new technique for the management of severe Asherman's syndrome	9591493
59	2004	47	Hysteroscopy in the evaluation of patients with recurrent pregnancy loss - A cohort study in a primary care population	15809790
60	2016	47	MicroRNA-29b inhibits endometrial fibrosis by regulating the Sp1-TGF-beta 1/Smad-CTGF axis in a rat model	26392347
61	1981	45	Significance of intrauterine adhesions detected hysteroscopically in eumenorrheic infertile women and role of antecedent curettage in their formation	7468688
62	1985	45	Etiology of cervical pregnancy. Association with abortion, pelvic pathology, IUDs and Asherman's syndrome	4038744
63	2008	44	Placenta accreta an association with fibroids and asherman syndrome	18946102
64	2014	44	Does cold loop hysteroscopic myomectomy reduce intrauterine adhesions? A retrospective study	24182410
65	2011	43	Outpatient hysteroscopy: a routine investigation before assisted reproductive techniques?	20638055
66	2013	43	Creation of a female rabbit model for intrauterine adhesions using mechanical and infectious injury	23199550
67	2012	42	Changes in endometrial receptivity in women with Asherman's syndrome undergoing hysteroscopic adhesiolysis	22535194
68	1994	41	Post-abortion-hysteroscopy--a method for early diagnosis of congenital and acquired intrauterine causes of abortions	7713291
69	2015	41	Results of centralized Asherman surgery, 2003-2013	26428306
70	1992	40	Hysteroscopic findings after missed abortion	1521644
71	1993	40	Induced regeneration of endometrium following curettage for abortion: a comparative study	8408501
72	2007	40	Fluoroscopically guided synechiolysis for patients with Asherman's syndrome: menstrual and fertility outcomes	17109860
73	1997	39	Successful treatment of severe uterine synechiae with transcervical resectoscopy combined with laminaria tent	9194644
74	2005	39	Pathologic findings in hysteroscopy before in vitro fertilization-embryo transfer (IVF-ET)	16316847
75	2014	39	Diagnostic accuracy of three-dimensional sonohysterography compared with office hysteroscopy and its interrater/intrarater agreement in uterine cavity assessment after hysteroscopic metroplasty	24581576
76	1989	38	Value of intrauterine device insertion and estrogen administration after hysteroscopic metroplasty	2549238
77	2008	38	Thin unresponsive endometrium-a possible complication of surgical curettage compromising ART outcome	18797990
78	1983	37	Diagnosis and treatment of intrauterine adhesions by microhysteroscopy	6825866
79	2017	37	Prevalence of intrauterine adhesions after the application of hyaluronic acid gel after dilatation and curettage in women with at least one previous curettage: short-term outcomes of a multicenter, prospective randomized controlled trial	28390688
80	2018	37	Allogeneic cell therapy using umbilical cord MSCs on collagen scaffolds for patients with recurrent uterine adhesion: a phase I clinical trial	29996892
81	2010	36	Results of 2500 office-based diagnostic hysteroscopies before IVF	20207586
82	2017	36	Reproductive outcomes in patients with intrauterine adhesions following hysteroscopic adhesiolysis: experience from the largest women's hospital in China	27856386
83	2017	36	Effects of Aspirin and intrauterine balloon on endometrial repair and reproductive prognosis in patients with severe intrauterine adhesion: a prospective cohort study	28251159
84	1976	35	Intrauterine adhesions secondary to elective abortion. Hysteroscopic diagnosis and management	
85	1992	35	Diagnostic hysteroscopy: its value in an in-vitro fertilization/embryo transfer unit	1291572
86	1996	35	Hysteroscopic management of uterine synechiae: a series of 102 observations	8730623
87	1999	35	Fluoroscopically guided hysteroscopic division of adhesions in severe Asherman syndrome	10362178
88	2000	35	Operative hysteroscopy for infertility using normal saline solution and a coaxial bipolar electrode: a pilot study	10920101
89	2007	35	Prevalence of uterine synechia after abortion evacuation curettage	18094891
90	2008	35	Genital tuberculosis: an important cause of Asherman's syndrome in India	17653564
91	2016	35	Endometrial stem cells repair injured endometrium and induce angiogenesis via AKT and ERK pathways	27486270
92	2015	34	Preventive effect of oral mucosal epithelial cell sheets on intrauterine adhesions	25475585
93	2007	33	Hysteroscopy after uterine fibroid embolization in women of fertile age	17578361
94	2016	33	The influence of the location and extent of intrauterine adhesions on recurrence after hysteroscopic adhesiolysis	25753391
95	2017	33	Human amniotic mesenchymal stromal cell transplantation improves endometrial regeneration in rodent models of intrauterine adhesions	28285950
96	1982	32	Asherman's syndrome. A comparison of therapeutic methods	7120210
97	1995	31	Preoperative sonographic measurement of endometrial pattern predicts the outcome of surgical repair in patients with severe Asherman's syndrome	7843453
98	1996	31	Intrauterine adhesions: detection with transvaginal US	8638001
99	1999	31	Total corporal synechiae due to tuberculosis carry a very poor prognosis following hysteroscopic synechialysis	10438408
100	1997	30	Simplified therapy for Asherman's syndrome	9418695

**Figure 4:**
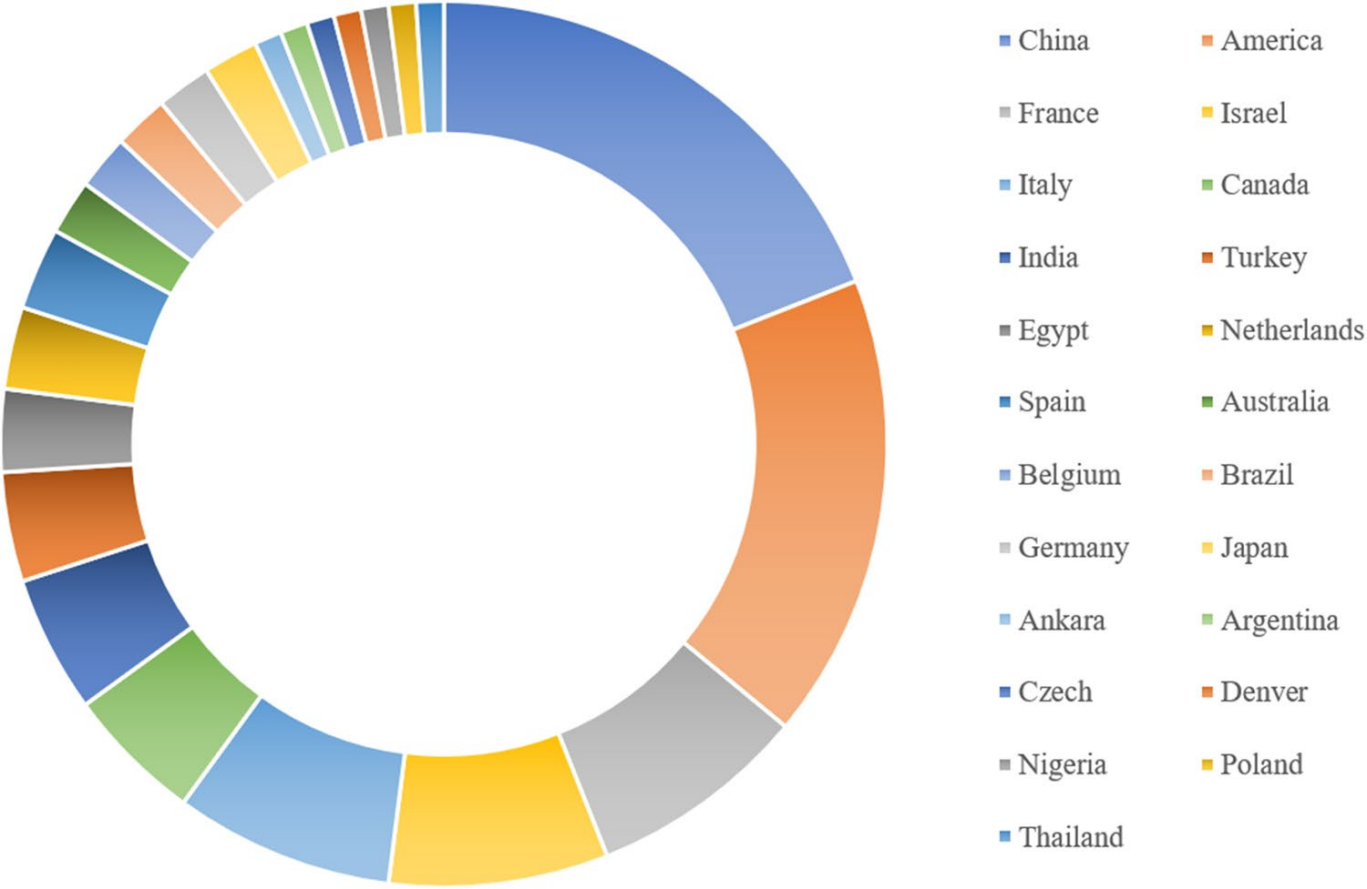
Country of origin of the top 100 most-cited articles in intrauterine adhesion

**Figure 5:**
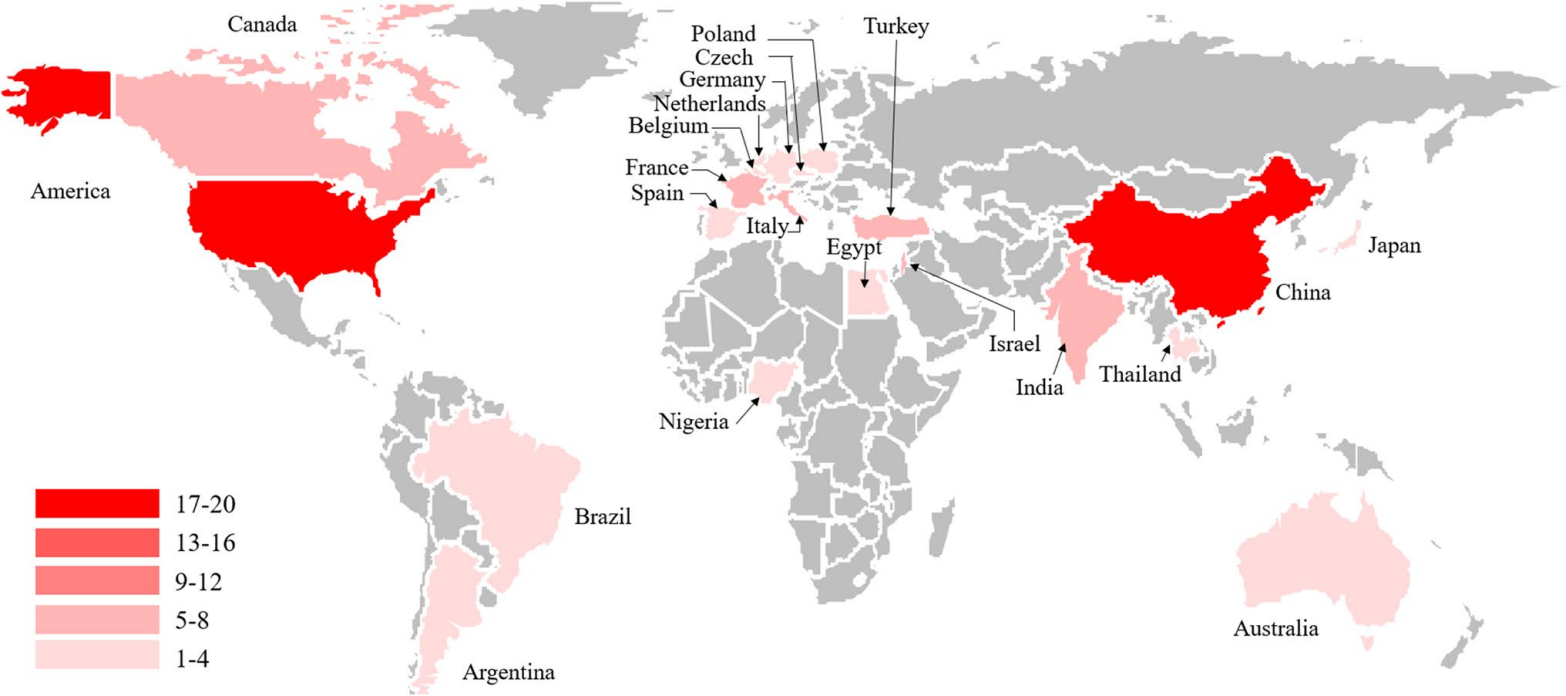
Geographical distribution of the 100 top most-cited articles in intrauterine adhesion

These articles were distributed in 22 countries (Fig. 4), led by China (*n*=19) and followed by the USA (*n*=18), France (*n*=8), Israel (*n*=8), and Italy (*n*=8). The allocation is presented on the world map (Fig. 5). In terms of regional distribution, most of the articles were published in two continents: North America and Asia. The other articles were scattered in Europe, Africa, and Oceania. Developed countries accounted for 67% of all countries mentioned above. In all, 33 journals accounted for all the articles published, among which 27 were published in *Fertility and Sterility*, 16 in *Human Reproduction*, 6 in *American Journal of Obstetrics and Gynecology*, and 5 in *Journal of Minimally Invasive Gynecology* (Table 2).

**Table 2:** Journals of the 100 top-cited studies published

Publication	Number account	Total citation	Average citation	IF (2020)
Fertility and Sterility	27	1895	70	6.3
Human Reproduction	16	1171	73	5.7
American Journal of Obstetrics and Gynecology	6	602	100	6.5
Journal of Minimally Invasive Gynecology	5	310	62	3.1
Obstetrics and Gynecology	4	187	47	5.5
Archives of Gynecology and Obstetrics	3	174	58	2.3
European Journal of Obstetrics, Gynecology, and Reproductive Biology	3	153	51	1.9
International Journal of Gynecology & Obstetrics	3	236	79	2.2
Journal of Obstetrics and Gynaecology Research	3	178	59	1.4
The Journal of Reproductive Medicine	3	115	38	0.2
Blog-An International Journal of Obstetrics and Gynaecology	2	97	49	4.7
Journal of Assisted Reproduction and Genetics	2	95	48	2.8
Reproductive Biomedicine Online	2	108	54	3.2
Ajr. American Journal of Roentgenology	1	58	58	3.0
American Journal of Roentgenology	1	62	62	3.0
Biomaterials	1	52	52	10.3
Biomed Research International	1	36	36	2.3
Contraception	1	51	51	2.8
Cytotherapy	1	33	33	4.2
Gynecological Endocrinology	1	39	39	1.6
International Journal of Clinical and Experimental Pathology	1	57	57	0.3
Journal of Human Reproductive Sciences	1	54	54	0.8
Journal of Clinical Ultrasound	1	117	117	1.0
Journal of Surgical Research	1	43	43	1.8
Journal of Ultrasound In Medicine	1	44	44	1.8
Journal of Womens Health	1	50	50	1.9
Jsls: Journal of The Society of Laparoendoscopic Surgeons	1	99	99	1.5
Plos One	1	114	114	2.7
Radiology	1	31	31	7.9
Reproduction	1	35	35	3.2
Reproductive Sciences	1	47	47	2.6
Sao Paulo Medical Journal	1	35	35	1.0
Stem Cell Research & Therapy	1	37	37	5.1
Surgical Endoscopy and Other Interventional Techniques	1	47	47	3.1

Among the authors of the 100 most cited articles, 10 had more than 2 articles (Table 3). These authors’ articles are all clinical studies related to IUA. Among them, March and Yang had 3 first authorships, mainly in the field of IUA. March’s focus was on hysteroscopic adhesiolysis, and Yang’s research direction was endometrial regeneration. Although Valle had only 2 articles, which were mainly focused on the auxiliary diagnosis of IUA, the number of citations was relatively high, with an overall number of citations of 286.

**Table 3:** List of first authors with frequent articles within the top-cited list

First Author	Number of Studies	Citations Account	Author's Affiliation
C M March	3	226	Department of Obstetrics and Gynecology, University of Southern California School of Medicine Los Angeles, California, USA.
Jehn-Hsiahn Yang	3	159	Department of Obstetrics and Gynecology, National Taiwan University Hospital and National Taiwan University College of Medicine, Taipei, Taiwan.
R F Valle	2	286	Department of Obstetrics and Gynecology, Northwestern University Medical School, Chicago, Illinois, USA
Mohamed I Amer	2	157	Department of Obstetrics and Gynecology, Ain Shams University, Cairo, Egypt.
Recai Pabuccu	2	153	Department of Obstetrics and Gynecology, Gülhane School of Medicine, Ankara, Turkey.
Xiaona Lin	2	125	Center of Reproductive Medicine, Sir Run Run Shaw Hospital, School of Medicine, Zhejiang University, PR China.
H Fernandez	2	113	Department of Obstetrics and Gynaecology, Antoine Béclère Hospital, Clamart Cedex, France.
Yuqing Chen	2	93	Department of Obstetrics and Gynecology, The First Affiliated Hospital of Sun Yat-sen University, Guangzhou, China.
P J Taylor	2	93	Department of Obstetrics and Gynecology, University of Calgary and Foothills Hospital, Calgary, Alberta, Canada
A Golan	2	75	Department of Obstetrics and Gynecology, Assaf Harofeh Medical Center, Zerifin, Israel.

**Figure 6:**
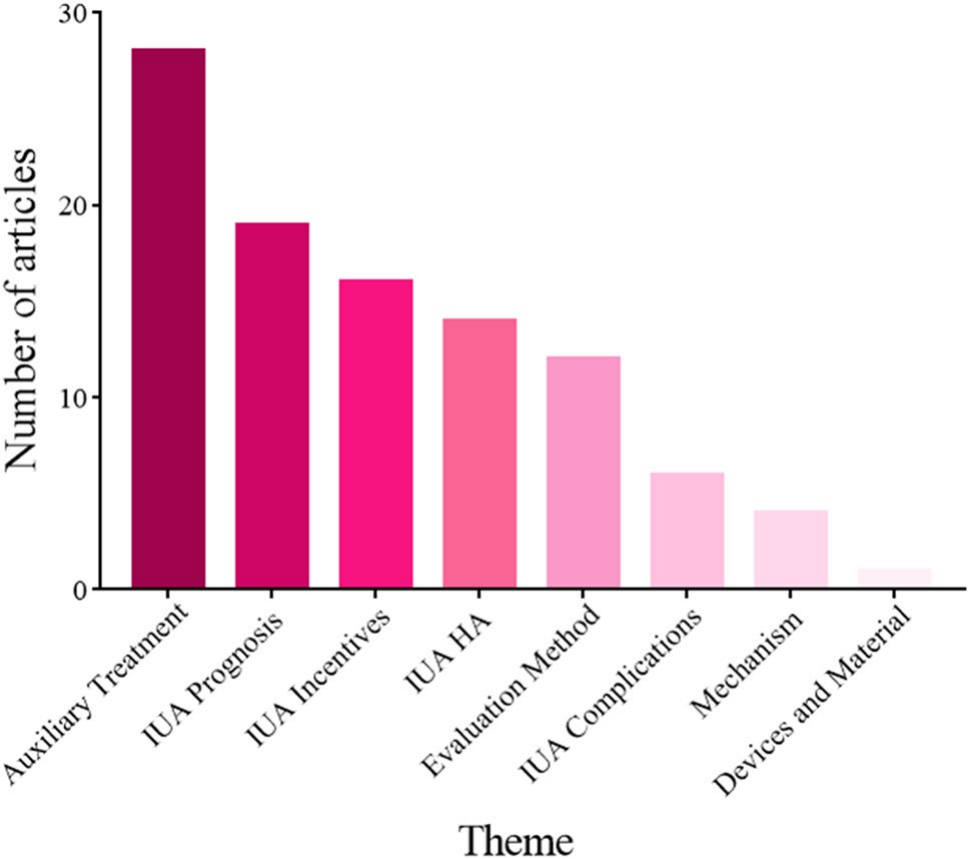
The thematic distribution of the 100 top most-cited articles in intrauterine adhesion

**Figure 7:**
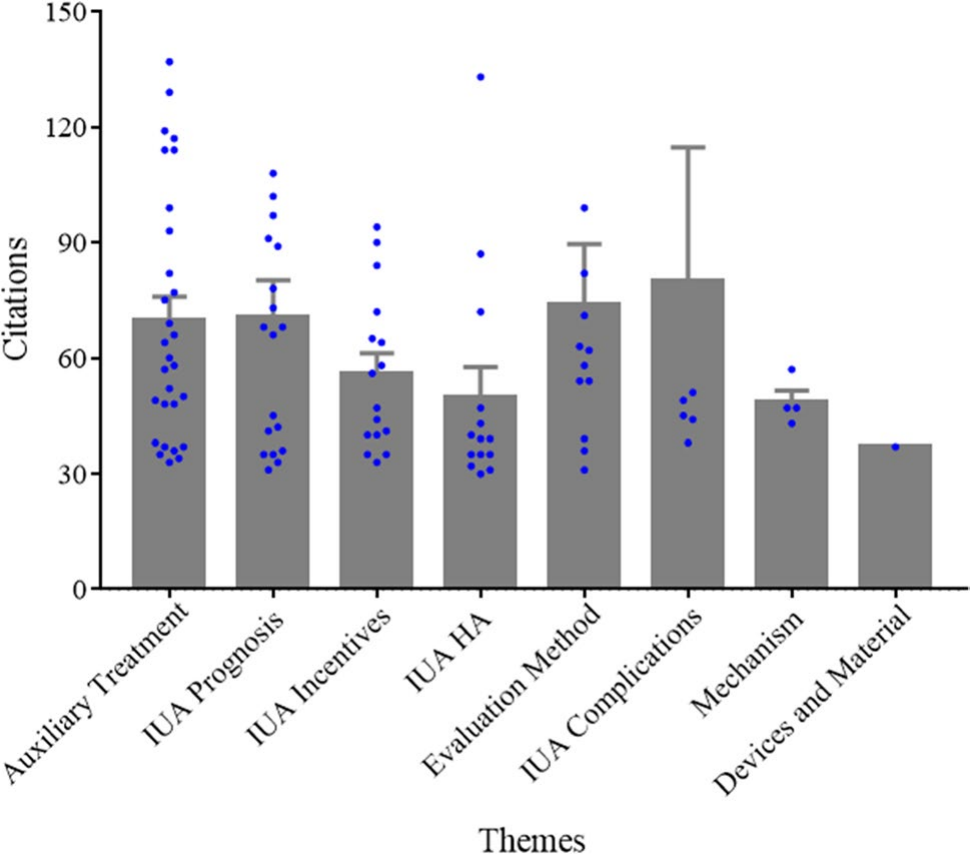
Mean citation per article based on the theme

**Figure 8:**
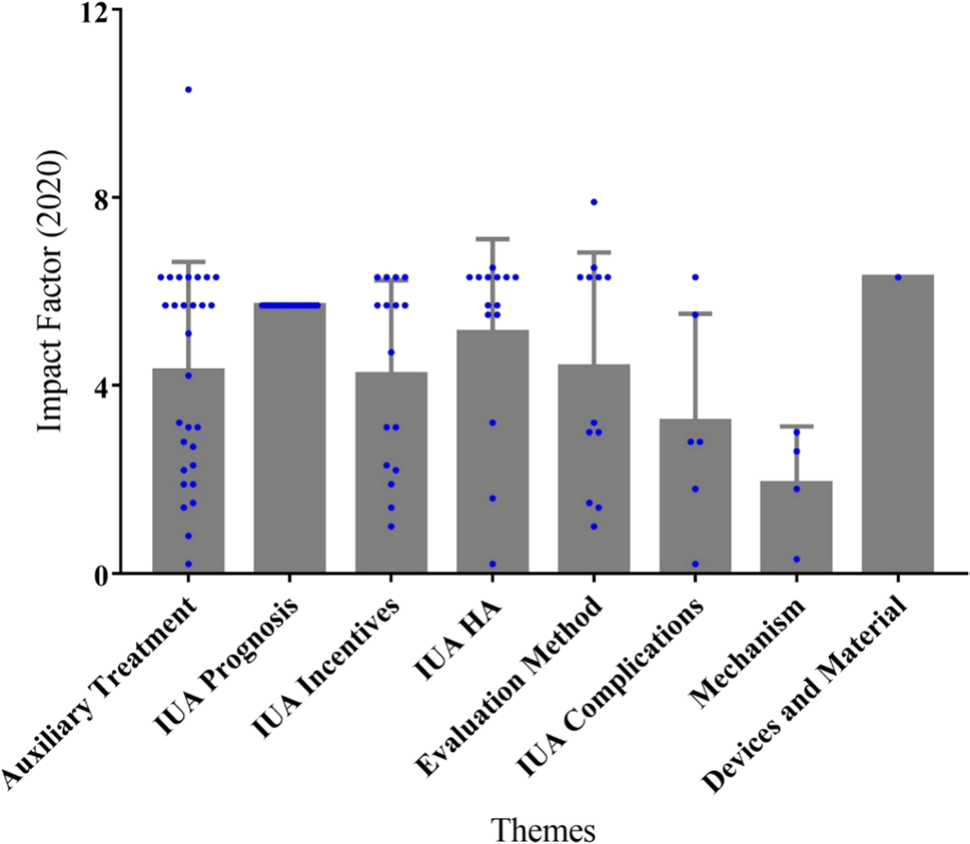
Mean impact factor per article based on the theme

The 100 most cited articles centered primarily on the following themes: auxiliary treatment of IUA (*n* = 28), the prognosis of IUA (*n*=19), incentive factors of IUA (*n*=16), hysteroscopic adhesiolysis (*n*=14), evaluation methods for IUA (*n*=12), complications of IUA (*n* = 6), relevant mechanism of IUA (*n* = 4), and new devices for IUA (*n*=1). The most frequently mentioned theme was the adjuvant treatment of IUA, followed by the reproductive prognosis of patients after hysteroscopic adhesiolysis (Fig. 6). One-way ANOVA revealed no significant difference in the citations per article across the various themes (Fig. 7). Nevertheless, the article’s impact factor from different themes was statistically different (*P*=0.012) (Fig. 8).

**Figure 9:**
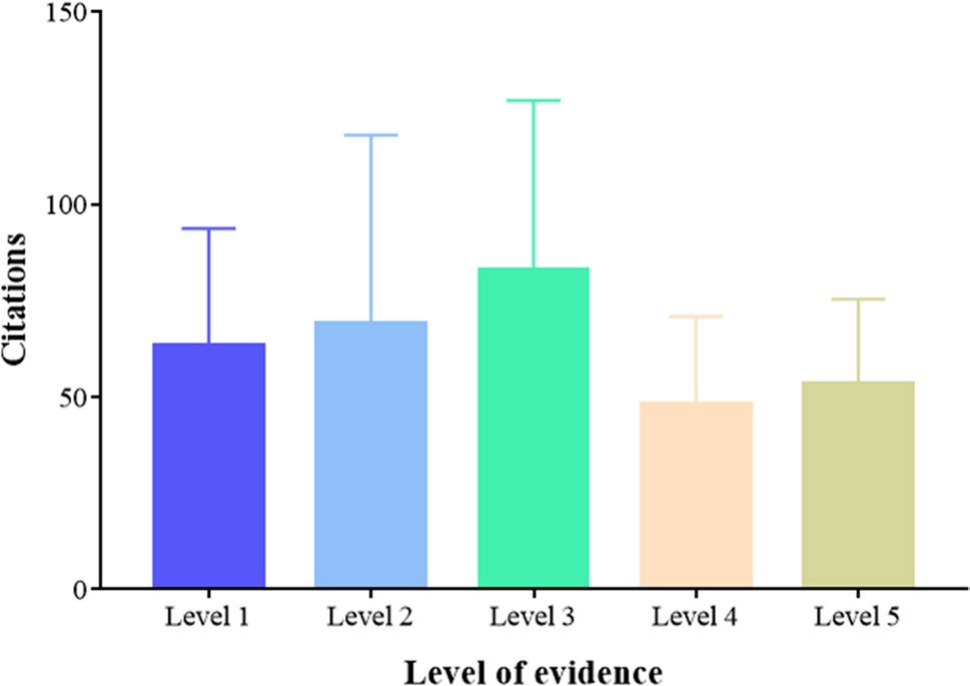
Mean citation per article based on level of evidence

In terms of the level of article evidence, the level II (*n*=39) category had a mean of 69 ± 48 citations per article, representing the largest number among the levels; the level I (*n*=23) category had a mean 63±30 citations per article, while level IV (*n*=14) had 48±22 citations. One-way ANOVA indicated that the differences in citations per article between different levels of evidence were not significant (Fig. 9).

**Figure 10:**
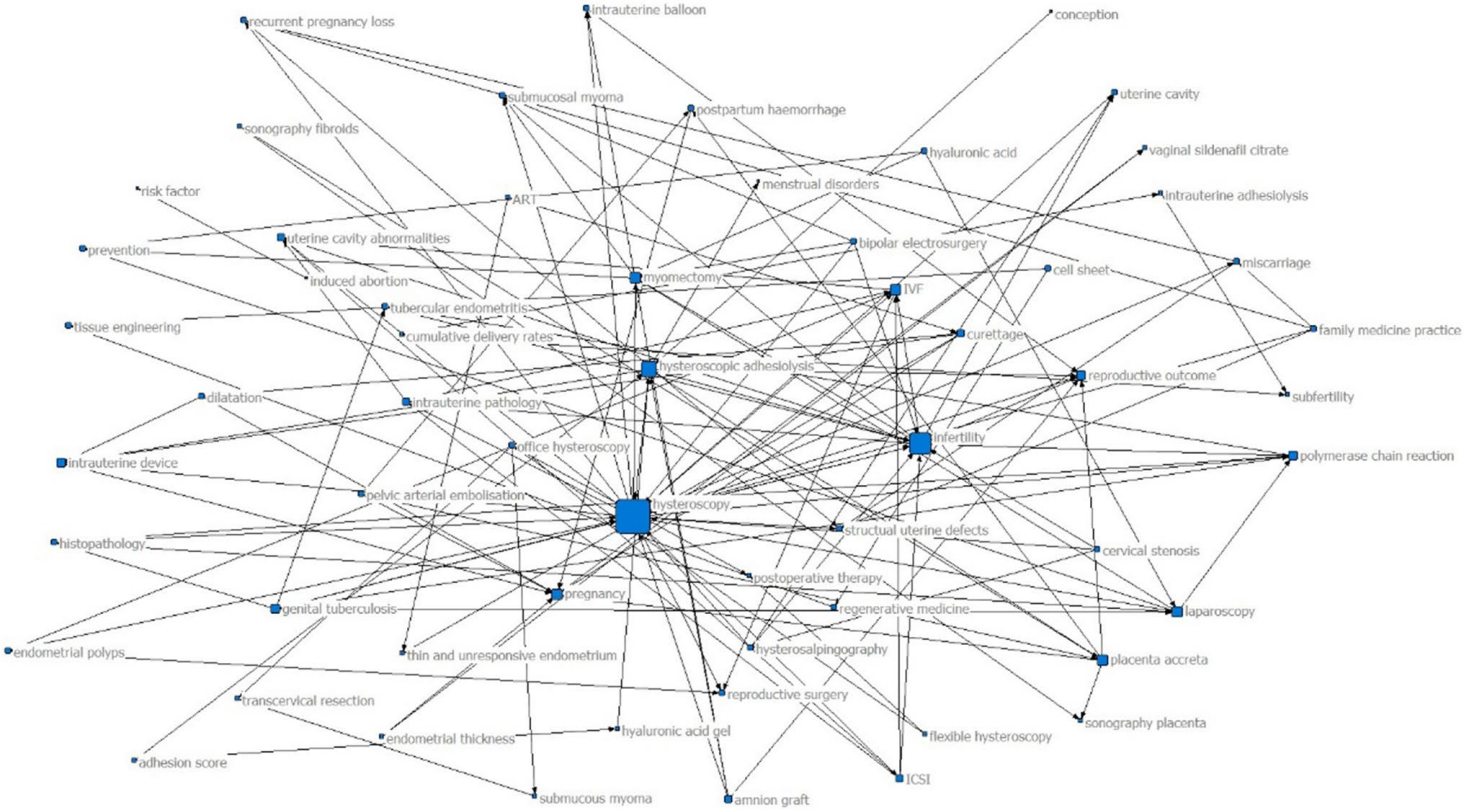
Degree centrality analysis in the 2000s (34 articles)

**Figure 11:**
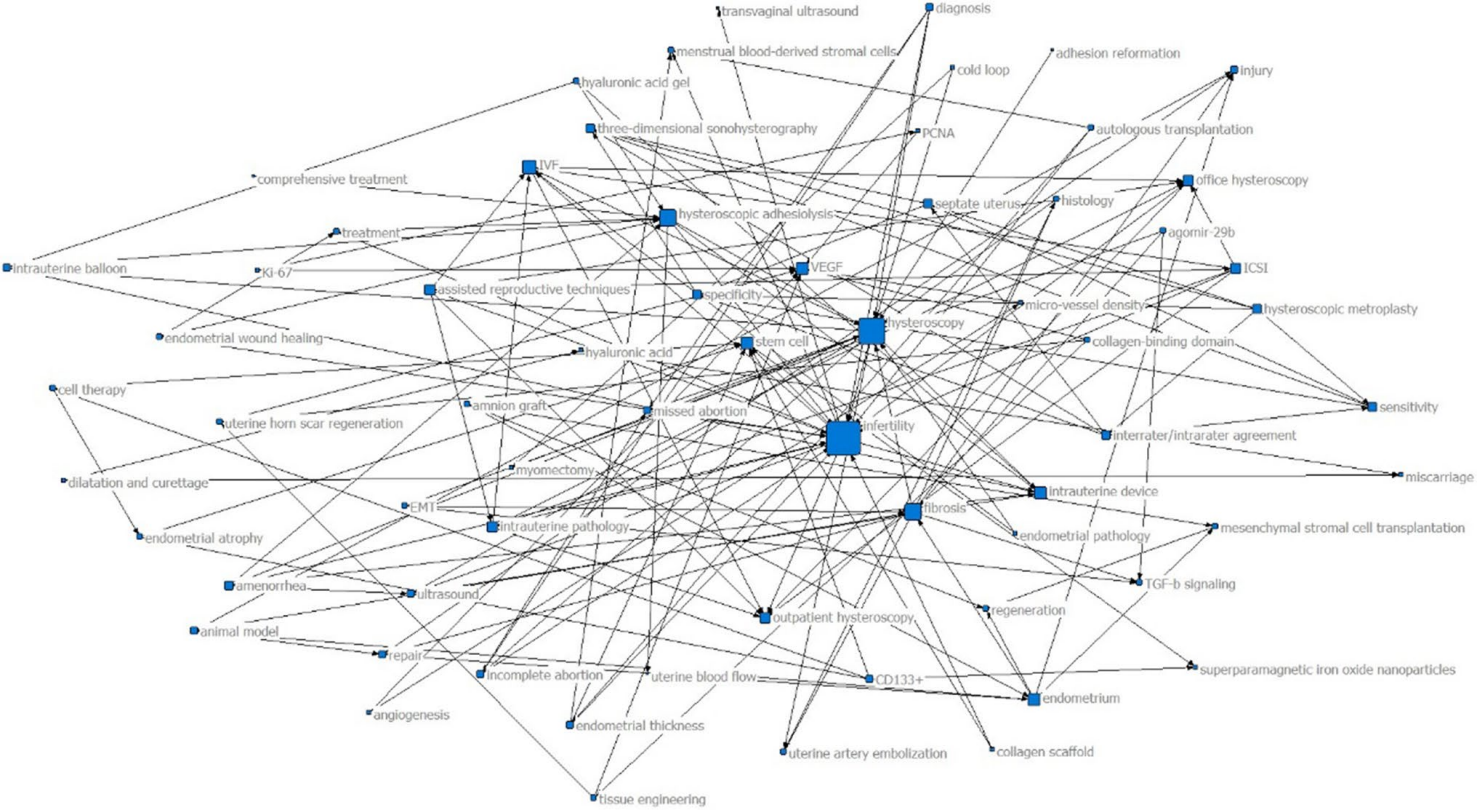
Degree centrality analysis in the 2010s (30 articles)

Network analysis of the keywords or subject terms was conducted in 2 periods of publication: in the 2000s (34 articles) and the 2010s (30 articles). The result indicated that “hysteroscopy”, “hysteroscopic adhesiolysis”, “infertility”, and “reproductive outcome” possessed a high degree of centrality in the 2000s and 2010s; “placenta accreta” was considerably centralized in the 2000s; and “stem cell” and “fibrosis” were highly concentrated in the 2010s (Figs. 10, 11).

## Discussion

This is the first bibliometric analysis of papers in the field of IUA. Several interesting findings can be drawn from this analysis of the top 100 most cited papers published on IUA over the past 70 years, which include insights into those papers which had made important contributions to the progress in the field and the current trends in development. Generally, it is expected that as time passes, the number of citations of papers would increase. However, contrary to this expectation, when we assessed the 100 most cited articles by decade, we found that articles published in the 2010s accounted for the highest proportion, reaching 35%; as time elapsed, both the numbers of articles and the citation density (i.e., number of citations divided by the number of years) grew. For one, this reflects the “obliteration by incorporation” [39] in the field, in which the citations of the original work decrease with time due to its familiarity and long-term, widespread use, coupled with the effect of replacement by novel findings. For another, this indicates that the diagnosis, treatment, and advanced research related to IUA have attracted more of the international community’s attention. In addition, 91% of these articles were clinical studies, and only 9% were basic experimental studies, with these basic studies all being published in the 2010s. Although clinical research has always been the focus of researchers in the field of IUA, understanding the disease mechanism is also crucial to the treatment of the disease. Recently, the number of studies published concerning the mechanism of IUA has been on the rise, and findings from these and similar studies may suggest novel directions for clinical research.

In contrast to the trend of other bibliometric analysis reports, articles originating from the USA and developed countries did not have the largest number of papers. China and the USA were both important sources of articles, publishing 19 and 18 articles, respectively. Furthermore, 33% of the 100 most cited papers were from developing countries, and 67% were from developed countries. There are several reasons for this: (1) The clinical manifestations of IUA were first reported in the USA in the nineteenth century and later defined and named by American doctors. Therefore, they were able to study it earlier and more systematically [40]. Furthermore, the USA and other developed countries had the requisite funding to support scientific research. The sharing of these research results will help research into IUA [41, 42]; (2) The occurrence of IUA is closely related to abortion and curettage, and abortion is illegal in many developing countries due to religious beliefs. In developed countries, the rate of abortion dropped by 19% from 1990 to 2014, while it only fell by 2% in developing countries over the same period [43]. Moreover, least-safe abortions accounted for the largest proportion of abortions in developing countries [44]. Thus, IUA is more likely to be an issue in developing countries, and such concern emerged as the research focus. (3) China and other developing countries have large populations. As the promotion and popularization of contraceptive measures in these populations are typically inadequate, unintended pregnancies and thus election of surgical abortion may be more common. Repeated or informal intrauterine operations can ultimately lead to irreversible damage to the endometrium [45, 46]. (4) After China introduced the 2-child policy, the incidence of IUA and the number of cases of recurrent IUA requiring treatment grew [47, 48]. IUA is a universal disease; however, nowhere in these 100 most cited papers was a multinational cooperative initiative mentioned.

Our results indicated that the journals that published the identified articles did not have high impact factors. This is likely because these papers were rarely published in comprehensive journals, and the impact factors of professional obstetrics and gynecology journals are relatively low. This result highlights a growing tendency of researchers perhaps preferring to publish articles in influential professional journals. However, there is no denying that the value of IUA research has not been fully appreciated.

Nearly 50% of the 100 papers were issued in 2 journals, *Fertility and Sterility* and *Human Reproduction*, followed closely by 2 prominent journals in the field of obstetrics and gynecology, *American Journal of Obstetrics and Gynecology* and *Obstetrics and Gynecology*. This demonstrates that IUA was of particular concern in the field of reproductive medicine. Among the 100 most cited articles, the themes of papers published in recent years appeared to be more diverse. They included, for example, a phase I clinical study of autologous stem cell transplantation for the treatment of IUA published in *Stem Cell Research & Therapy* [49] and a collagen scaffold for endometrial regeneration study published in *Biomaterials* [50].

Among the authors of the 100 most cited papers, we found that 2 researchers contributed 3 articles. One of these authors was Charles M. March from the USA. His articles were all published before 1990 and emphasized hysteroscopy as the gold standard for the diagnosis and classification of IUA and hysteroscopic adhesiolysis as the first choice for the treatment of IUA [51–53]. The other author was Jehn-Hsiahn Yang from Taiwan, China, who is from a younger generation of researchers. The similarity with March was a focus on hysteroscopy. Yang’s 3 articles asserted that IUA is a common complication after transcervical resection of multiple apposing submucous myomas [54], the formation of new adhesions after hysteroscopic adhesiolysis affects endometrial repair [55], and the location and area of adhesions are important factors that affect the recurrence of IUA [15].

Among the 100 articles, the level of evidence was between I and V. The overall distribution was average, with level II being relatively high, which is different from other bibliometric studies. This may be attributable to the design of clinical trials. Treatment of IUA is closely related to patients’ reproductive prognosis, and for women, age is also a significant factor. Therefore, considering the effect of placebo control on patients, experimental studies are mostly unilateral. In the future, with the increase in the demand for childbirth and the introduction of 2 or even 3-child policies in some countries, it is expected that the rate of research into hysteroscopic adhesiolysis, postoperative adjuvant treatment, and related subjects will rapidly increase.

We also studied the thematic distribution of articles. The adjuvant treatment of IUA ranked first, followed by the prognosis of reproductive outcome after hysteroscopic adhesiolysis, the incentive factors of IUA, hysteroscopic adhesiolysis, and the evaluation method of IUA. Hysteroscopic adhesiolysis is the primary treatment method for IUA. However, how to improve the therapeutic effect through drugs and biological agents after the surgery has been a problem that researchers have sought to overcome in recent years [56]. Network analysis of the authors’ keywords or subject terms was conducted in 2 periods of publication: in the 2000s (34 articles) and the 2010s (30 articles). The result indicated that “hysteroscopy”, “hysteroscopic adhesiolysis”, “infertility”, and “reproductive outcome” were highly centralized in both 2000s and 2010s, and “placental implantation” was highly concentrated in the 2000s. This was consistent with the research trend in recent years. However, the topic of IUA prevention presented a blank state in the highly cited papers, indicating that breakthroughs in early prevention are urgently needed in this field.

Researchers have gradually realized that even if patients with IUA succeed in conception, there remain many risks of pregnancy complications. The most cited article in this study was also an article about IUA complications, in which the author proposed that patients with IUA were associated with many risks during pregnancy. In analyzing the citation density of papers published in the 2010s, we found that each theme was evenly distributed. In analyzing the impact factor under each theme, it could be seen that articles related to devices and materials were relatively high, while research on mechanisms was relatively low. Stem cell and fibrosis were highly concentrated in the 2010s. In recent years, researchers have paid increasing attention to the effectiveness of adjuvant therapy after hysteroscopic adhesiolysis in suppressing the reformation of adhesion and promoting endometrial regeneration [57]. Previous research focused on the placement of uterine stents, Foley catheters, hyaluronic acid, or hormone replacement therapy [58]. Nevertheless, more importance was attached to biotherapy, especially in the field of stem cell therapy. Bone marrow mesenchymal stem cells (BMSCs) [59], umbilical cord mesenchymal stem cells (UCMSCs) [60], and menstrual blood mesenchymal stem cells (MbMSCs) [61, 62] have been used clinically in patients with IUA, resulting in an improvement in reproductive outcome. Furthermore, researchers reported that stem cells could not only promote endometrial cell proliferation, but also inhibit the process of fibrosis [63].

In 2020, a review gathered the controversial views of different researchers on the treatment of IUA [64]. One perspective advocated that the adjuvant treatment after the surgery should be given more attention, and another suggested that IUA be treated with multiple operations. However, hysteroscopic adhesiolysis has always been the center of research over the years, and discussions concerning surgical methods and surgical techniques are still sparse [65]. It is undeniable that combined adjuvant treatment after surgery is the dominant trend of IUA treatment.

This analysis does generate some valuable information, but some limitations should be mentioned. First, the citation analysis was mainly based on the Web of Science, and the number of citations may be misleading. Meanwhile, we might have missed some important papers that were not included in the Science Citation Index database. Second, we might have overlooked the newly published articles that were meaningful in this area but have yet to reach high citation levels due to the criterion that the number of citations sorted papers. Overall, our analysis was by no means exhaustive, but the list of most cited articles still includes many influential papers in the field of IUA.

## Conclusion

This article highlights the top 100 most cited articles in IUA research published over the last 70 years, including their publication time and regional centers, first author, level of evidence, and research theme. Furthermore, our findings support the notion that postoperative adjuvant therapy has played a key role in the field of IUA, and more and more basic and clinical studies are now exploring suitable and effective adjuvant treatments for IUA. Meanwhile, the method and techniques of surgical therapy have been controversial. Regardless, the current prognosis of IUA remains extremely poor, and many difficult challenges need to be overcome. Researchers have concentrated their focus on clinical trials rather than on basic laboratory research. Thus, the foremost difficulty lies in addressing the limited understanding of the underlying mechanisms of adhesion formation in the uterus and the other influencing factors.

## Data Availability

Not applicable

## Abbreviations

IUA: Intrauterine adhesion
IUD: Intrauterine device
ACP: Auto-crosslinked hyaluronic acid
BMSC: Bone marrow mesenchymal stem cell
UCMSC: Umbilical cord mesenchymal stem cell
MbMSC: Menstrual blood mesenchymal stem cell
